# Is hypertensive left ventricular hypertrophy a cause of sustained ventricular arrhythmias in humans?

**DOI:** 10.1038/s41371-021-00503-w

**Published:** 2021-03-05

**Authors:** R. Nadarajah, P. A. Patel, M. H. Tayebjee

**Affiliations:** Department of Cardiology, Leeds General Infirmary, Leeds, UK

**Keywords:** Ventricular tachycardia, Risk factors

## Abstract

Sudden cardiac death (SCD) is most commonly secondary to sustained ventricular arrhythmias (VAs). This review aimed to evaluate if left ventricular hypertrophy (LVH) secondary to systemic hypertension in humans is an isolated risk factor for ventricular arrhythmogenesis. Animal models of hypertensive LVH have shown changes in ion channel function and distribution, gap junction re-distribution and fibrotic deposition. Clinical data has consistently exhibited an increase in prevalence and complexity of non-sustained VAs on electrocardiographic monitoring. However, there is a dearth of trials suggesting progression to sustained VAs and SCD, with extrapolations being confounded by presence of co-existent asymptomatic coronary artery disease (CAD). Putatively, this lack of data may be due to the presence of more homogenous distribution of pathophysiological changes seen in those with hypertensive LVH versus known pro-arrhythmic conditions such as HCM and myocardial infarction. The overall impression is that sustained VAs in the context of hypertensive LVH are most likely to be precipitated by other causes such as CAD or electrolyte disturbance.

## Introduction

Systemic hypertension is a major public health problem with recent estimates implicating it as the cause of around 7.5 million deaths globally per annum [1]. It is an established risk factor for congestive heart failure (CHF), coronary artery disease (CAD) and cerebrovascular disease [2]. Sudden cardiac death (SCD) is the most serious clinical manifestation of cardiac disease. In most instances, SCD relates to the occurrence of sustained ventricular arrhythmias (VAs), particularly ventricular tachycardia (VT) and fibrillation (VF) [3]. Patients with chronic, systemic hypertension are known to develop left ventricular hypertrophy (LVH) secondary to adverse ventricular remodelling. However, it is unclear whether hypertensive LVH is an isolated risk factor for ventricular arrhythmogenesis.

## Aims and hypothesis

The aim of this review was to assess whether systemic hypertension in combination with LVH is an independent risk factor for VAs in those without established CAD. We hypothesised that hypertensive heart disease on its own does not contribute to the risk of VAs or SCD. We therefore reviewed the published literature on hypertension and VAs and critically appraised the data to determine whether hypertensive LVH on its own causes VAs.

## Search strategy

MEDLINE (inception to 10th October 2020), Embase (inception to 10th October 2020), Oxford Academic (to 10th October 2020) and Google Scholar (to 10th October 2020) were searched using a priori database-appropriate MESH terms relating to ‘hypertension’, ‘left ventricular hypertrophy’, ‘arrhythmia’, ‘ventricular arrhythmias’, ‘ventricular fibrillation’, ‘ventricular tachycardia’ and ‘sudden cardiac death’. Both animal and human studies were eligible for inclusion. All types of study were included for assessment including reviews, systematic reviews, meta-analyses and original research (case series, case-control studies, cohort studies and randomised controlled trials). Articles in languages other than English were excluded.

From this initial search, duplicate records were removed, and the remainder screened for suitability based on title and abstract independently by acknowledged contributors BSK and AS and then combined. Potentially suitable studies were then viewed in full text by BSK, AS and author RN independently and relevance for inclusion was decided by either unanimous agreement or, when there was disagreement, adjudicated by RN. Derived references from these sources were used to seek other potentially relevant citations. The final choice of included studies was then validated by authors PP and MHT.

## Basic science data

**Fig. 1:**
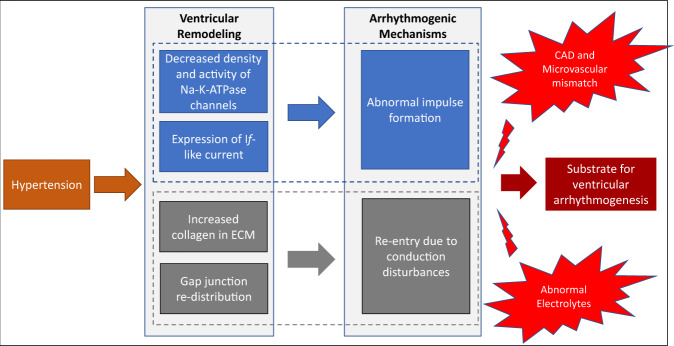
Postulated mechanisms underpinning ventricular arrhythmias in hypertensive left ventricular hypertrophy. ECM *Extracellular matrix,* CAD *Coronary artery disease*.

The most common pattern of left ventricular (LV) remodelling in hypertension is concentric hypertrophy, reflecting increased LV mass and relative wall thickness. At a cardiomyocyte level, this arises from assembly of contractile protein units in parallel, resulting in increased width of individual myocytes. These alterations are accompanied by changes in ion channels, extracellular matrix, gap junctions and microvasculature, which could play a role in ventricular arrhythmogenesis in hypertensive LVH (see Fig. 1).

### Changes in ion channels

In vitro animal models of hypertensive LVH have shown a number of changes in the density and function of ion channels in ventricular myocytes. Histological analysis of spontaneously hypertensive rat hearts have observed reductions in the density and activity of the sodium–potassium–adenosine triphosphatase (Na + K + ATPase) pump at the sarcolemma; this could affect the stability of the resting membrane potential and theoretically predispose to VAs [4]. Another study has observed that 90% of isolated ventricular myocytes in spontaneously hypertensive rats can express an I*f*-like current that may be activated at voltages near the physiological diastolic potential and produce abnormal, spontaneous cell depolarisation (i.e. heightened automaticity) [5]. Similarly, a murine model of hypertensive LVH using whole-cell patch-clamp recordings found that potassium current densities during repolarisation were significantly lower when compared with controls [6]. This was associated with a more prolonged QTc interval and action potential duration (APD), which may provide substrate for development of early (EADs) or delayed afterdepolarisations (DADs) that can precipitate VAs. Moreover, non-uniform prolongation that is characteristically associated with LVH may be pro-arrhythmic by increasing dispersion of repolarisation or refractoriness and favouring conditions for re-entry mechanisms that may result in VAs [7].

In vitro studies of human hypertrophied cardiac myocytes have also demonstrated changes in ion channels. One study observed that afterload-induced hypertrophy was associated with messenger RNA upregulation and concurrent protein expression of a non-cardiac voltage-gated sodium channel (Na_V_1.8). Using a whole-cell patch-clamp technique, inhibition of these channels with novel blockers reduced late and persistent sodium current and shortened APD. In addition, this current precipitated spontaneous calcium release in diastole, secondary to leak from the sarcoplasmic reticulum, which can also give rise to DADs and therefore VAs [8].

### Fibrosis and gap junctions

There is increased collagen synthesis in cultured ventricular myocytes in response to hypertension [9]. The presence of fibrosis may create a milieu that leads to dispersion of refractoriness, heterogeneity of electrical conduction and re-entry circuit formation. Spontaneously hypertensive rats had higher cardiac mass and fibrotic burden, which was associated with higher burden of VAs (defined as an isolated ventricular ectopic beat or more) and suppressed with trandolapril therapy [10]. In humans, simple regression analysis confirmed a strong correlation between hypertrophy, fibrosis and VAs; however, analysis was confined to VAs collectively without differentiation between sustained and non-sustained dysrhythmias; additionally, the correlation was present only for severe hypertrophy. In patients with established LVH, those with known VAs had more subendocardial fibrosis when assessed by endomyocardial biopsy; notably, there was no similar correlation with co-existent CAD or CHF albeit VAs were limited to those that were non sustained [11].

Pressure-overloaded hearts in aortic-banded rats have a more heterogeneous distribution of Connexin-43 (Cx43) gap junctions throughout the ventricular myocardium [12]. This may conceivably lead to slower inter-cellular conduction, heightening the probability of micro re-entry circuit formation and VAs. In a separate rat model, reduction in Cx43 gap junction density was associated with increased susceptibility to sustained VAs in the context of low extracellular potassium [13]. This notion is supported by a double-transgenic rat model, in which re-institution of normal channel localisation and upregulation of Cx43 gap junction expression, through use of n-3 polyunsaturated fatty acid (PUFA) ethyl-esters, reduced QRS and QTc intervals and increased the threshold required to induce VAs by programmed electrical stimulation [14].

### Microvasculature

Murine models have shown that the cardiac microvasculature cannot sustain metabolic demands associated with hypertrophy [15]. This may result in deleterious effects on cellular vascular endothelial growth factor signalling due to attenuation of the transcription factor HIF-1α [16, 17]. This mismatch between cellular demand and vascular supply can lead to micro-ischaemic areas even in the absence of epicardial CAD. However, these pathological perturbations are driven primarily by chronic ischaemia from under-perfusion rather than hypertension per se. These chronic changes may produce a more arrhythmogenic milieu if epicardial CAD does develop. For example, dogs with hypertensive LVH undergoing left coronary artery occlusion had higher propensity for sustained monomorphic VT (41% vs. 6%; *p* < 0.05) and reperfusion-associated VF (88% vs. 0%; *p* < 0.05) compared to those that were normotensive; this disparity occurred despite infarct size being comparable across groups [18, 19].

## Clinical data

It is challenging to dissect the relative contributions of hypertensive LVH and concurrent ischaemia on susceptibility to VAs. In humans, significant epicardial CAD, defined as coronary stenosis >50% on invasive coronary angiography, is present in up to 40% of asymptomatic hypertensive patients with LVH and recorded non-sustained VT [11]. Ischaemia arising from macrovascular disease is an established trigger for VAs even when asymptomatic, as demonstrated by one study observing higher burden of non-sustained VAs in those with co-existent thallium-201 perfusion defects (14% vs. 4%; *p* < 0.05) [20]. In hypertensive patients without manifest CAD, a close temporal relationship has been shown between the occurrence of VAs and episodes of transient ST depression, which were predictive for future cardiac events including SCD [21]. However, it remains unclear whether these two risk factors precipitate each other or are instead covariate responses to an increased sympathetic drive.

Pertinently, although ventricular ectopy is of common prevalence in the general population, it does not necessarily translate to progression into sustained VAs and heightened risk of SCD. Furthermore the extent of fibrosis in subjects with hypertensive LVH also does not match that occurring in other specific cohorts. For instance, in autopsies of those suffering SCD, ventricular myocardium from patients with hypertrophic cardioymyopathy (HCM) had threefold higher deposition of matrix collagens than hypertensive comparators and exhibited a more disorganised pattern [22].

Data from clinical studies broadly suggest that the degree of hypertrophy must be marked to increase susceptibility to VAs. In two studies, where CAD had been excluded by invasive angiography, there was no increase in frequency of VAs at mild to moderate levels of LVH [23, 24]. Another study utilised endocardial catheter mapping and observed higher prevalence of left ventricular late potentials in patients with hypertensive LVH, which correlated with detection of VAs on ambulatory monitoring [25]. However, this trend was observed almost exclusively in those with decompensated hypertensive heart disease, i.e. with concurrent CHF, rather than those with isolated systemic hypertension.

The observed QTc prolongation that arises in hypertensive LVH through changes in expression and distribution of ion channels does, however, appear to have clear correlations with arrythmogenesis. QTc duration appears to be related to left ventricular mass index and the most prolonged QTc intervals were detected in those with LVH and complex VAs [26]. Another study found that prolonged QTc duration predicted mortality risk in patients with ECG criteria for LVH, being highest in those with QTc > 500 ms [27]. Beyond prolongation, associated dispersion of the QTc interval is an index of inhomogeneity of repolarisation and has been strongly linked with vulnerability to sustained VAs [28].

Data from observational studies does not show a convincing link between hypertensive LVH and SCD secondary to sustained VAs. One study in the Framingham population showed that the presence of an echocardiographic diagnosis of LVH in hypertensive patients was associated with an increased risk of SCD (hazard ratio 2.16, 95% CI 1.22–3.81, *p* = 0.008) [29]. However it is unclear from this data what proportion of excess events were related to sustained VAs, and during the follow-up period 28% of those with LVH were diagnosed with MI prior to SCD suggesting that ischaemia could have contributed to the adverse prognosis [30]. Furthermore a separate study from the Framingham population showed that in hypertensive patients with an echocardiographic diagnosis of LVH the presence of complex or frequent ventricular ectopy (≥class 2 on Lown grading system) on 1 h ECG recordings was associated with a marginally significant increase in all-cause mortality (hazard ratio 1.62). However it is not commented what proportion of these excess events were SCD or if any of them related to sustained VAs. Furthermore again these patients were had a higher incidence of myocardial infarction during follow-up, whilst at the start of follow-up, CAD had only been ruled out by lack of symptoms, rather than invasive coronary angiogram. Finally a meta-analysis of LVH and VAs has been conducted that incorporates five studies and 5659 patients [31]. Results were suggestive of a 2.8 fold greater risk of VT or VF in the presence of hypertensive LVH, and without significant inter-study heterogeneity (*I*^2^ = 9%). However, all included studies were observational with varying study designs and patient characteristics (Table 1). Two studies were confounded by co-existent CAD [32, 33], and three other studies only demonstrated non-sustained VAs [34**–**36].

**Table 1:** Summary of studies incorporated into meta-analysis [4] assessing the association between hypertensive left ventricular hypertrophy and ventricular arrhythmias.

Study	Ref.	Study design	Population	Patients	Hypertension (%)	LVH diagnosis	Patients with LVH		Patients without LVH		Confounder
							With documented VAs	Total	With documented VAs	Total	
Levy et al.	[34]	Observational	Framingham, USA	4960	NR	ECG/Echo	6	843	12	3994	Only asymptomatic NSVT, no sustained VAs
McLenachan et al.	[35]	Observational	Framingham, USA	150	67	ECG	14	50	5	100	Only asymptomatic NSVT, no sustained VAs
Novo et al.	[33]	Observational	Palermo, Italy	128	100	Echo	8	66	2	62	VA prevalence correlated with increasing TEMI
Bender et al.	[32]	Observational	New York, USA	317	NR	ECG	31	109	33	208	All patients had known ischaemic cardiomyopahy

*ECG* electrocardiograph, *LVH* left ventricular hypertrophy, *NR* not reported, *NSVT* non-sustained ventricular tachycardia, *TEMI* transient episode of myocardial ischaemia (St segment downsloping >1 mm, 80 ms after J point, with a duration of almost 1 min on 24 h holter monitoring), *VAs* ventricular arrhythmias, *VEs* ventricular ectopic beats, *VF* ventricular fibrillation, *VT* ventricular tachycardia.

Trials that have looked at the benefit of LVH regression by pharmacotherapy have not shown a causal link to sustained VAs. Meta-analysis has shown that angiotensin II receptor antagonists, angiotensin-converting enzyme inhibitors, calcium antagonists, diuretics and beta blockers can induce regression of LVH to varying degrees [37]. The HOPE (Heart Outcomes Prevention Evaluation) trial showed that prevention/regression of ECG-diagnosed LVH was associated with a reduction in cardiovascular death, cardiac arrest myocardial infarction and stroke. However these effects were seen in all sub-groups, including without hypertension, and hint that some of this effect may be related to renin–angiotensin–aldosterone inhibition rather than blood pressure modification [38]. In addition the LIFE (Losartan Intervention For Endpoint Reduction in Hypertension) trial showed that angiotensin II blockade was superior compared to atenolol at reducing morbidity and mortality in hypertensive patients alongside reduction in LVH [39]. However this was primarily driven by a 25% relative risk reduction in stroke with no difference in cardiovascular death. Furthermore one trial showed that treatment with ramipril ± felodipine would reduce left ventricular mass index but not produce a sustained reduction in ventricular ectopic beats. Thus pharmacotherapy can improve outcomes in hypertensive patients but this does not appear to be by prevention of sustained VAs [40].

Overall, there appears to be a dearth of trials confirming association between hypertensive LVH and progression to sustained VAs and SCD. By contrast, there are several other pathological conditions either associated with systemic hypertension or resulting in increased LV mass index that have been validated to increase dysrhythmic risk (Table 2) [41]. The reason for the disparity for sustained VAs between animal models and human clinical data, as well as between hypertensive LVH and other conditions resulting in increased LV mass such as HCM, may be due to the homogenous distribution of changes seen in hypertensive LVH. Late gadolinium enhancement on cardiac magnetic resonance imaging has shown that fibrosis in hypertensive LVH patients is predominantly non-subendocardial. In patients with HCM, it is primarily non-subendocardial and typically distributed anteroseptally, inferoseptally and at right ventricular insertion points with these focal changes linked to increased prevalence and complexity of VAs on ambulatory monitoring [42, 43]. Another study has shown that focal asymmetrical hypertrophy in HCM, compared to the more diffuse distribution seen in hypertensive LVH, was inversely correlated with QTc dispersion time in V1–V4 and these patients had a higher risk of sustained VT on subsequent implantable cardioverter-defibrillator interrogations [44]. The combination of structural and electrical remodelling (involving gap junctions and ion channels) alongside focal fibrosis appears to result in increased transmural dispersion of repolarisation and secondary preponderance to sustained VAs.

**Table 2:** Conditions associated with hypertension or increased LV mass index that have evidence for increased risk of sustained ventricular arrhythmias.

**Condition**
Coronary artery disease
Left ventricular systolic dysfunction
Hypertrophic cardiomyopathy
Amyloidosis
Anderson–Fabry disease
Cardiac sarcoidosis

## Conclusion

The pathophysiological changes found in animal models of hypertensive LVH, including cellular changes (ion channels) and abnormalities in inter-cellular conduction (fibrosis and gap junction re-distribution), provide a putative basis for ventricular arrhythmogenesis in this population. Clinical data in humans has shown an increased prevalence and complexity of VAs in hypertensive LVH patients but there is a lack of confirmatory trial data suggestive of progression to sustained VAs that can cause SCD. This may be due to the more homogenous distribution of pathophysiological changes seen in hypertensive LVH when compared with known pro-arrhythmic disorders such as HCM and myocardial infarction where there is myocardial disarray and/or fibrosis.
